# Maternal lipid levels in early pregnancy as a predictor of childhood lipid levels: a prospective cohort study

**DOI:** 10.1186/s12884-022-04905-7

**Published:** 2022-07-23

**Authors:** Maria C. Adank, Anja K. Johansen, Laura Benschop, Sophia P. Van Streun, Anna M. Smak Gregoor, Linn K. L. Øyri, Monique T. Mulder, Eric A. P. Steegers, Kirsten B. Holven, Jeanine E. Roeters van Lennep

**Affiliations:** 1 Department of Obstetrics and Gynaecology, Erasmus MC, University Medical Centre Rotterdam, Rotterdam, the Netherlands; 2 Generation R Study Group, Erasmus MC, University Medical Centre Rotterdam, Rotterdam, the Netherlands; 3 Department of Nutrition, Institute of Basic Medical Sciences, University of Oslo, Oslo, Norway; 4 National Advisory Unit on Familial Hypercholesterolemia, Oslo University Hospital, Oslo, Norway; 5 Department of General Medicine, Erasmus MC, University Medical Centre Rotterdam, Rotterdam, the Netherlands

**Keywords:** Pregnancy, Lipoproteins, HDL, Triglycerides, Cholesterol, LDL

## Abstract

**Background:**

Maternal lipid levels in early pregnancy are associated with maternal health and foetal growth. It is however unclear if maternal lipids in early pregnancy can be used to predict childhood lipid levels. The aim of this study is to assess the association between maternal and offspring childhood lipid levels, and to investigate the influence of maternal BMI and diet on these associations.

**Methods:**

This study included 2692 women participating in the Generation R study, an ongoing population-based prospective cohort study from early life onwards. Women with an expected delivery date between 2002 and 2006 living in Rotterdam, the Netherlands were included. Total cholesterol, triglycerides and high-density lipoprotein cholesterol (HDL-c) were measured in early pregnancy (median 13.2 weeks [90% range 10.6; 17.1]). Low-density lipoprotein cholesterol (LDL-c), remnant cholesterol and non-HDL-c were calculated. Corresponding lipid measurements were determined in 2692 children at the age of 6 (median 6.0 years [90% range 5.7; 7.5]) and 1673 children 10 years (median 9.7 years [90% range 9.5; 10.3]). Multivariate linear regression analysis was used to examine the association between maternal lipid levels in early pregnancy and the corresponding childhood lipid measurements at the ages of 6 and 10 years while adjusting for confounders.

**Results:**

Maternal lipid levels in early pregnancy are positively associated with corresponding childhood lipid levels 6 and 10 years after pregnancy, independent of maternal body mass index and diet.

**Conclusions:**

Maternal lipid levels in early pregnancy may provide an insight to the lipid profile of children years later. Gestational lipid levels may therefore be used as an early predictor of children’s long-term health. Monitoring of these gestational lipid levels may give a window-of-opportunity to start early interventions to decrease offspring’s lipid levels and possibly diminish their cardiovascular risk later in life. Future studies are warranted to investigate the genetic contribution on maternal lipid levels in pregnancy and lipid levels of their offspring years later.

**Supplementary Information:**

The online version contains supplementary material available at 10.1186/s12884-022-04905-7.

## Background

In pregnancy the maternal metabolism undergoes adaptations to support maternal and foetal demands. Regarding lipoprotein metabolism these changes consist of increased levels of total cholesterol, low-density lipoprotein cholesterol (LDL-c), high-density lipoprotein cholesterol (HDL-c), and in particular triglycerides during gestation [1]. The prevalence of hyperlipidaemia in pregnancy is still unknown. Maternal lipid levels are mainly determined by genetics and lifestyle factors such as diet and obesity. Maternal lipid levels in early pregnancy are associated with pregnancy complications such as pre-eclampsia and gestational diabetes [2–4]. Recently, gestational lipid levels were also found to be associated with metabolic syndrome many years after pregnancy, suggesting that the lipid profile during pregnancy may be used as an early marker of women’s cardiovascular health in later life [5]. The influence of maternal lipid levels in pregnancy on health markers in their children is less studied. High levels of triglycerides and remnant cholesterol in early pregnancy have been associated with adverse neonatal outcomes such as increased risk of a child born large-for-gestational age [6, 7]. In addition, foetuses and children of mothers with elevated cholesterol levels show more and larger fatty streak formation in their aortas, and a more rapid progression to atherosclerosis in early childhood [8, 9]. Balder et al. showed that the prevalence of hypercholesterolemia in children and adolescents of Caucasian descent in the Netherlands is 1:450 [10]. However, it is still unsure whether the maternal lipid profile in pregnancy may also provide a glimpse into long-term cardiovascular health of children. Therefore, the aim of this study was to assess the association between the lipid profile of women in early pregnancy with the lipid profile of children at the age of 6 and 10 years.

It is known that maternal lifestyle factors, such as an unhealthy diet and obesity, are associated with an increased cardiovascular risk in children [11, 12]. We therefore additionally examined the influence of body mass index and diet on the association of maternal lipid levels in pregnancy with corresponding lipid levels in children. Moreover, since lipid levels in children may be affected by foetal programming, we repeated all analyses in women without a placental syndrome (pre-eclampsia, a child born small-for-gestational age and spontaneous preterm birth) to minimize its effect [13].

## Methods

### Design and study population

**Fig. 1:**
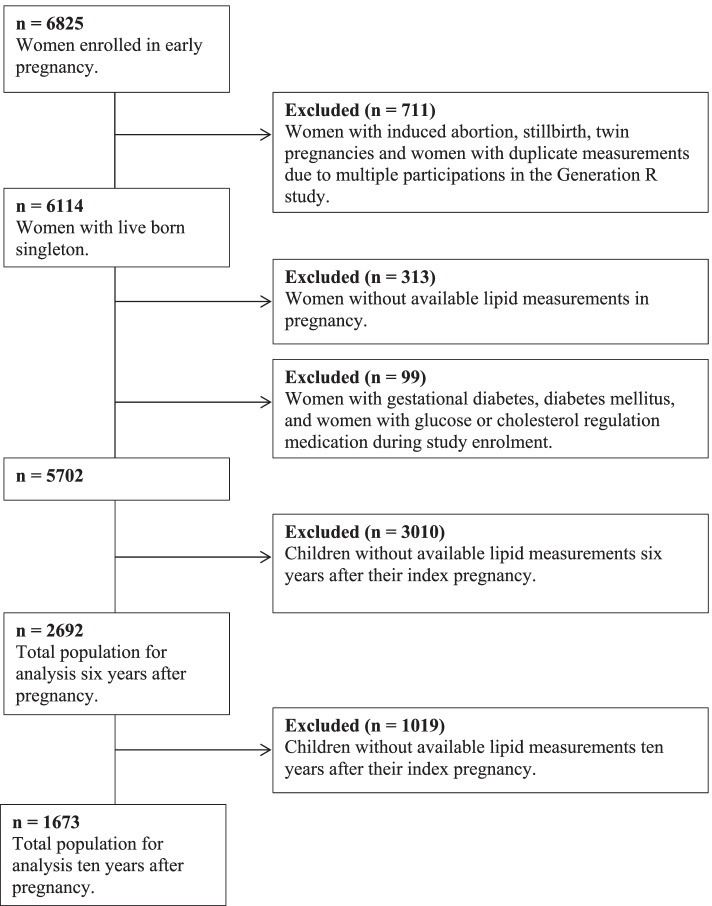
Flowchart

This study was embedded in the Generation R Study, an ongoing population-based prospective cohort study from early pregnancy onward in Rotterdam, the Netherlands [14]. Women enrolled between April 2002 and January 2006. All methods were performed in accordance with the relevant guidelines and regulations. For this study, we included women with a live born singleton and available information on lipid measurements in early pregnancy. We excluded women with a twin pregnancy, (gestational) diabetes mellitus, and women on glucose or lipid regulating medication at enrolment. Children without available lipid measurements at the different time points (6 and 10 years after pregnancy) were also excluded (Fig. 1). Additional file 2 contains a Strengthening the Reporting of Observational Studies in Epidemiology (STROBE) statement for the current study [15].

### Ethics approval and consent to participate

The study has been approved by the Medical Ethical Committee of the Erasmus Medical Centre in Rotterdam (MEC-2007-413). For minors, written informed consent from a parent and/or legal guardian was obtained. From the rest of the participants written informed consent was obtained.

### Exposure: maternal lipid levels

Non-fasting blood samples were obtained at enrolment in early pregnancy (median 13.2 weeks [90% range 10.6; 17.1]) by trained research nurses to determine the levels of total cholesterol, triglycerides, and high-density lipoprotein cholesterol (HDL-c) [16]. Total cholesterol, triglycerides and HDL-c were analysed using standard laboratory methods. The mean interassay coefficients of variation for total cholesterol, triglycerides, and HDL-c were, respectively, 4.2, 3.4, and 2.8%. Levels of LDL-c were calculated using the Friedewald equation [17]. Non-HDL-c was calculated by subtracting HDL-c from the total cholesterol and remnant cholesterol as ([total cholesterol – LDL-c] – HDL-c). Details of processing procedures and lipid calculations have been described previously [2, 7, 18].

### Placental syndrome

Placental syndrome was defined as having pre-eclampsia, a child born small-for-gestational age (SGA) or a spontaneous preterm birth (sPTB) in the index pregnancy. We obtained information on clinically diagnosed pre-eclampsia from medical records that were cross-checked with the original hospital charts [19]. Pre-eclampsia was defined, using the ISSHP criteria that were in effect at the time of the study, as the development of systolic blood pressure (SBP) ≥ 140 mmHg and/or a diastolic blood pressure (DBP) ≥ 90 mmHg with new-onset proteinuria in a random urine sample and no evidence of a urinary tract infection [20]. Midwife and hospital registries provided information on gestational age at birth, birth weight, and child’s sex. SGA was defined as a child with a birth weight below the 10th percentile, adjusted for gestational age and sex of the child. We defined sPTB as the spontaneous onset of labour before 37 weeks of gestation.

### Outcome: childhood lipid levels

Non-fasting venous blood samples were collected to measure total cholesterol, triglycerides and HDL-c in the children at the age of 6 (median 6.0 years [90% range 5.7; 7.5]), and 10 years (median 9.7 years [90% range 9.5; 10.3]). Blood samples were obtained, transported, and stored as described in detail previously [16]. Serum concentrations of total cholesterol, triglycerides and HDL-c were measured at the Erasmus Medical Centre with enzymatic methods (Cobas 8000, Roche, Almere, the Netherlands) [16, 21, 22]. Levels of LDL-c, remnant cholesterol and non-HDL-c were calculated [17].

### Covariates

Information on maternal characteristics during pregnancy including age, ethnicity, educational level, parity, smoking, and the use of cholesterol or glucose regulating medication, and information on smoking and gravidity 6 and 10 years after pregnancy was obtained through questionnaires. We obtained information on pre-pregnancy weight through questionnaires at study enrolment. Maternal weight (kilograms) and height (centimetres) were measured at enrolment in early pregnancy and 6 and 10 years after pregnancy without shoes and heavy clothing, after which body mass index (BMI) was calculated (kilograms per square meter). Pre-pregnancy weight and measured weight at enrolment were highly correlated (Pearson’s correlation coefficient 0.97 [value of *P* < .001]), and therefore, pre-pregnancy weight was used to calculate BMI in the analyses. At the age of 6 and 10 years, weight and height were measured without shoes and heavy clothing. We calculated BMI, and categorized children into age- and sex specific groups; normal-weight, overweight and obese, using the definition of Cole et al. [23]. Women’s dietary intake in early pregnancy was assessed using a semi-quantitative 293-item food frequency questionnaire (FFQ) at enrolment. The FFQ included foods that were frequently consumed in the Dutch population and was modified for use during pregnancy. National dietary guidelines were used to develop a predefined diet quality score for pregnant women. The diet score included 15 components in which the scores for the individual components were summed, resulting in an overall score ranging from 0 to 15, with a higher score representing a healthier diet [24].

### Statistical analyses

**Fig. 2:**
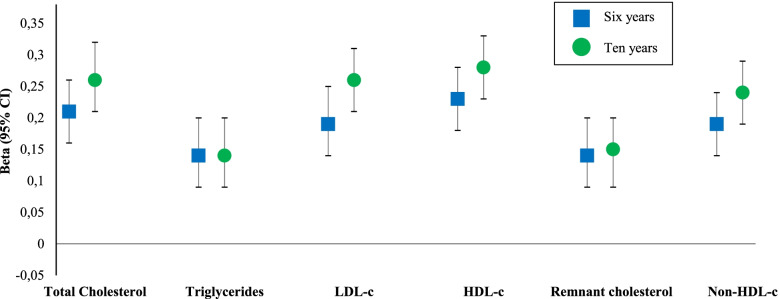
Association of maternal lipid profile in early pregnancy with childhood lipid levels 6 and 10 years after pregnancy. Abbreviations: BMI, body mass index; CI, confidence interval; LDL-c, low-density lipoprotein cholesterol; HDL-c, high-density lipoprotein cholesterol. Data were adjusted for maternal age at enrolment, gestational age at blood sampling, ethnicity, parity, educational level and pre-pregnancy BMI. Values are linear regression coefficients (95% confidence interval)

First, we describe pregnancy and follow-up characteristics for all women and children. The mean ± standard deviation is presented for data with a normal distribution and the median with 90% range for data with a skewed distribution. Second, we examined the distribution of early pregnancy lipid levels in women, and in their children at the age of 6 and 10 years. Third, we present the lipid distributions in children stratified on child’s sex. Paired sample *t* test and Wilcoxon signed rank test were used for comparison of paired samples. Independent samples were tested through Students *t* test and Mann-Whitney U test. Fourth, triglyceride and remnant cholesterol levels were log transformed to achieve a normal distribution. To enable comparison of effect estimates, we constructed SD-scores (SDS) for all lipid levels [7]. We tested whether the association of maternal lipid levels with corresponding childhood lipid levels was nonlinear. Fifth, since we found a linear relation, we further examined the association between maternal lipid levels in early pregnancy and the corresponding childhood lipid measurements at the age of 6 and 10 years using multiple linear regression models. These models were adjusted for maternal age at enrolment, gestational age at blood sampling, ethnicity, parity, educational level and pre-pregnancy BMI. Sixth, missing data of the covariates were imputed. We used the Markov Chain Monte Carlo multiple imputation procedures to reduce potential bias attributable to missing data [25]. In this study, 1.3% had missing information on ethnicity, 4.8% on educational level, 0.5% on parity, 9.6% on smoking in early pregnancy, 16.6% on pre-pregnancy BMI. For the regression analysis, we used the SDS for all lipid levels as exposure. Regression models adjusted for confounders were: basic model (maternal age at enrolment, gestational age at blood sampling, educational level, ethnicity, parity and smoking), BMI model (basic model additionally adjusted for pre-pregnancy BMI) and maternal diet model (BMI model additionally adjusted for diet score). These confounders were selected based on previous studies, and on their associations with the exposures and outcomes of interest. The effect estimates in Fig. 2 are regression coefficients from the BMI model and represent an increase in the outcome measure for each unit increase in the exposure. Seventh, we tested for unmeasured confounding through the E-value method (Additional file 1, Table S1) [26]. Eight, we tested whether there was effect modification in all associations by sex of the child, through inclusion of the interaction term (exposure * sex of child) in each regression model. Since we found no effect modification for the associations, we did not stratify the results on child’s sex. In addition, child’s sex did not contribute to our models, therefore we chose not to include child’s sex as a confounder. Ninth, in attempt to exclude the effect of placental syndromes, we performed the regression analyses in a subgroup of women without placental syndromes in their index pregnancy. Lastly, for clinical purposes we defined the 90th percentile for maternal gestational lipid levels (total cholesterol, triglycerides, LDL-c, remnant cholesterol and non-HDL-c), whereas for HDL-c we defined the 10th percentile. Thereafter we tested the association of these cut-off values with the corresponding lipid measurements in their children. Statistical analyses were performed with SPSS version 21.0 for Windows (SPSS INC, Chicago, IL, USA).

## Results

### General characteristics

This study included 2692 mothers with lipids measured in early pregnancy and children with lipids measured at 6 years. Ten years after pregnancy, lipid measurements were available in 1673 children (Fig. 1). Women were on average 30.3 (SD 4.9) years at the start of pregnancy (Table 1). They were mostly European, highly educated and nulliparous. Their pre-pregnancy BMI was 23.7 (90% range 19.5; 32.9) kg/m^2^ and 26.6% of the women smoked during their pregnancy. At the age of 6 years (median 6.0 years, 90% range 5.7; 7.5) children’s BMI was 15.9 (90% range 14.0; 19.4) and at the age of 10 years (median 9.7 years, 90% range 9.5; 10.3) children’s BMI was 16.9 (90% range 14.4; 22.9).

**Table 1:** Maternal and childhood characteristics

**Pregnancy**	***n*** **= 2692**
Maternal age (years)	30.3 (4.9)
Gestational age at blood sampling (weeks)	13.2 (10.6; 17.1)
Non-European ethnicity, n (%)	988 (36.7)
Education level, n (%)	
None/primary school	245 (9.1)
Secondary school	1163 (43.2)
Higher education	1284 (47.7)
Nulliparous, n (%)	1676 (62.3)
Pre-pregnancy BMI (kg/m^2^)	22.9 (18.8; 32.0)
Normal or underweight, n (%)	1895 (70.4)
Overweight, n (%)	582 (21.6)
Obesity, n (%)	215 (8.0)
Smoking during pregnancy, n (%)	715 (26.6)
Diet score pregnancy	7.7 (4.9; 10.2)
Gestational age at birth (weeks)	40.1 (37.1; 42.0)
Birth weight (g)	3435 (545)
Small-for-gestational age, n (%)	268 (10.0)
Spontaneous preterm birth, n (%)	88 (3.3)
Pre-eclampsia, n (%)	54 (2.1)
Male sex of child, n (%)	1361 (50.6)
**Follow-up 6 years after pregnancy**	***n*** **= 2692**
Maternal age (years)	37.0 (4.8)
Primigravid, n (%)	188 (9.4)
Maternal BMI (kg/m^2^)	24.6 (19.8; 35.4)
Normal or underweight, n (%)	1400 (53.4)
Overweight, n (%)	792 (30.2)
Obesity, n (%)	432 (16.5)
Smoking, n (%)	361 (18.4)
Age of child (years)	6.2 (0.6)
BMI child (kg/m^2^)	15.9 (14.0; 19.4)
Normal or underweight, n (%)	2221 (82.5)
Overweight, n (%)	360 (13.4)
Obesity, n (%)	111 (4.1)
LDL-c of child ≥3.5 mmol/L	100 (3.7)
**Follow-up 10 years after pregnancy**	***n*** **= 1673**
Maternal age (years)	41.1 (4.6)
Primigravid, n (%)	104 (6.9)
Maternal BMI (kg/m^2^)	24.7 (20.0; 35.1)
Normal or underweight, n (%)	864 (53.4)
Overweight, n (%)	478 (29.5)
Obesity, n (%)	276 (17.1)
Age of child (years)	9.8 (0.3)
BMI child (kg/m^2^)	16.9 (14.4; 22.9)
Normal or underweight, n (%)	1375 (82.3)
Overweight, n (%)	237 (14.2)
Obesity, n (%)	58 (3.5)
LDL-c of child ≥3.5 mmol/L	47 (2.8)

Data are presented as mean (SD) for continuous variables with a normal distribution, as median (90% range) for continuous variables with a skewed distribution or as n (%) for categorical variables. Women with a BMI < 25.0 kg/m^2^ were defined as normal or underweight, a BMI of 25.0–30.0 kg/m^2^ was considered as overweight and women with a BMI ≥ 30.0 kg/m^2^ were defined as obese. Childhood underweight, normal weight, overweight, and obesity were defined by the International Obesity Task Force cutoffs [23]

*Abbreviations*: *BMI* Body mass index, *LDL* Low-density lipoprotein cholesterol

### Maternal and offspring lipid levels

Table 2 shows the lipid profile of women in early pregnancy and the lipid profile of children at the age of 6 and 10 years. BMI, total cholesterol, triglycerides, HDL-c, and remnant cholesterol levels are significantly higher, while LDL-c and non-HDL-c levels are significantly lower at the age of 10 years than at the age of 6 years. Additional file 1, Table S2 shows the lipid distribution in children stratified by child’s sex. At 6 and 10 years, girls have a more atherogenic lipid profile with higher levels of total cholesterol, triglycerides, LDL-c, remnant cholesterol, non-HDL-c, and lower levels of HDL-c than boys.

**Table 2:** Lipid profile of women in early pregnancy and children at the age of 6 (*n* = 2692) and 10 years (*n* = 1673)

Lipids levels	Early Pregnancy (*n* = 2692)	Child 6 years (n = 2692)	Child 10 years (*n* = 1673)	***P***-value
Age, years	30.3 (4.9)	6.2 (0.6)	9.8 (0.3)	< 0.001
BMI, kg/m^2^	22.9 (18.8; 32.0)	15.9 (14.0; 19.4)	16.9 (14.4; 22.9)	< 0.001
Total cholesterol, mmol/L	4.82 (0.88)	4.21 (0.64)	4.31 (0.64)	< 0.001
Triglycerides, mmol/L	1.25 (0.71; 2.38)	0.95 (0.46; 2.04)	0.99 (0.48; 2.34)	< 0.001
LDL-c, mmol/L	2.42 (0.73)	2.38 (0.59)	2.32 (0.58)	< 0.001
HDL-c, mmol/L	1.79 (0.35)	1.34 (0.31)	1.46 (0.34)	< 0.001
Remnant cholesterol, mmol/L	0.57 (0.33; 1.08)	0.43 (0.21; 0.93)	0.45 (0.22; 1.06)	< 0.001
Non-HDL-c, mmol/L	3.03 (0.83)	2.86 (0.62)	2.84 (0.63)	0.02

Data are presented as mean (SD) for continuous variables with a normal distribution or as median (90% range) for continuous variables with a skewed distribution. Differences between measurements of children at the age of 6 and 10 years were tested through Paired sample t-test or Wilcoxon Signed rank test

*Abbreviations*: *LDL-c* Low-density lipoprotein-cholesterol, *HDL-c* High-density lipoprotein-cholesterol

### Association between maternal gestational and offspring lipid levels at 6 and 10 years

Maternal total cholesterol, triglyceride, LDL-c, HDL-c, remnant cholesterol and non-HDL-c levels are all positively associated with the corresponding measurements in children at the age of 6 and 10 years (Fig. 2). These results are independent of maternal pre-pregnancy BMI and diet (Fig. 2 and Table 3). In addition, the results of our regression analyses were not influenced by placental syndrome since the results were similar when the analyses were repeated in a subset of women without placental syndrome in their index pregnancy (*n* = 2143) (Additional file 1, Table S3). In Additional file 1, Table S4 we show the association of cut-off values of maternal lipid levels in early pregnancy with the corresponding childhood lipid measurements. We found that all levels were positively associated, independent of confounders.

**Table 3:** Association of maternal lipid profile in early pregnancy with their corresponding offspring lipid level at the age of 6 and 10 years

**Exposure** **Lipids in pregnancy**	**Outcome** **Lipid levels of children 6 years (*n*** **= 2692)**		
	**Basic model** Beta (95% CI)	**Maternal BMI model** Beta (95% CI)	**Maternal diet model** Beta (95% CI)
Total cholesterol, SDS	0.21 (0.16; 0.26)	0.21 (0.16; 0.26)	0.21 (0.16; 0.26)
Triglycerides, SDS	0.14 (0.09; 0.19)	0.14 (0.09; 0.20)	0.14 (0.09; 0.20)
LDL-c, SDS	0.19 (0.14; 0.25)	0.19 (0.14; 0.25)	0.19 (0.14; 0.25)
HDL-c, SDS	0.23 (0.18; 0.28)	0.23 (0.18; 0.28)	0.23 (0.18; 0.28)
Remnant cholesterol, SDS	0.14 (0.09; 0.19)	0.14 (0.09; 0.20)	0.14 (0.09; 0.20)
Non-HDL-c, SDS	0.19 (0.14; 0.24)	0.19 (0.14; 0.24)	0.19 (0.14; 0.24)
**Lipids in pregnancy**	**Outcome** **Lipid levels of children 10 years (*n*** **= 1673)**		
	**Basic model** Beta (95% CI)	**Maternal BMI model** Beta (95% CI)	**Maternal diet model** Beta (95% CI)
Total cholesterol, SDS	0.26 (0.21; 0.32)	0.26 (0.21; 0.32)	0.26 (0.21; 0.32)
Triglycerides, SDS	0.15 (0.10; 0.21)	0.14 (0.09; 0.20)	0.14 (0.09; 0.20)
LDL-c, SDS	0.26 (0.21; 0.31)	0.26 (0.21; 0.31)	0.26 (0.21; 0.31)
HDL-c, SDS	0.29 (0.24; 0.34)	0.28 (0.23; 0.33)	0.28 (0.23; 0.33)
Remnant cholesterol, SDS	0.16 (0.10; 0.21)	0.15 (0.09; 0.20)	0.15 (0.09; 0.20)
Non-HDL-c, SDS	0.25 (0.20; 0.30)	0.24 (0.19; 0.29)	0.24 (0.19; 0.29)

Values are regression coefficients reflecting the difference in childhood lipid level with 95% confidence interval derived from multiple linear regression analyses. Basic model: adjusted for maternal age at intake, gestational age at blood sampling, ethnicity, parity, smoking and educational level. Maternal BMI model: basic model additionally adjusted for pre-pregnancy BMI. Maternal diet model: BMI model additionally adjusted for maternal diet

*Abbreviations*: *BMI* Body mass index, *CI* Confidence interval, *SDS* SD-scores, *LDL-c* Low-density lipoprotein cholesterol, *HDL-c* High-density lipoprotein cholesterol

## Discussion

This study shows that maternal lipid levels in early pregnancy are positively associated with lipid levels of children at ages 6 and 10 years. These associations were independent of maternal pre-pregnancy BMI and maternal diet.

Previous studies on the associations of lipid levels measured in pregnancy with lipid levels in children are mostly limited to total cholesterol, LDL-c, HDL-c, and triglycerides. This study shows that all measured gestational lipid levels, including remnant cholesterol and non-HDL-c, are positively associated with lipid levels in children, independent of maternal pre-pregnancy BMI and diet. This is in agreement with findings from the Framingham Heart Study cohort demonstrating that elevated maternal LDL-c before pregnancy was correlated to offspring LDL-c in young adulthood (mean age 26 years); while interestingly, paternal LDL-c was not associated with the offspring LDL-c [27]. A study from the Rhea pregnancy cohort also showed a positive association in 348 mother/child pairs between gestational total cholesterol and LDL-c with total cholesterol of children at the age of four, independent of maternal pre-pregnancy BMI [28]. Similarly, a smaller study by Christensen et al. that included women in early pregnancy (gestational week 14 to 16) showed that 27 women with LDL-c levels in the upper percentiles during early gestation had offspring with significantly higher LDL-c levels (0.4 mmol/L) at the age of 6–13 years, compared to 34 women with LDL-c in the lower percentiles [29]. A study by Juhola et al. found a strong relationship between childhood lipid levels and lipid levels measured in middle age [30], which underlines the importance of early markers for the cardiovascular disease risk of children in childhood and thereafter.

Gestational lipids in early pregnancy may be associated with childhood lipids through four potential pathways. First, we hypothesized that lifestyle factors would largely explain the association between gestational lipid levels and lipid levels of children years later. However, the associations between maternal and offspring total cholesterol, triglycerides, LDL-c, HDL-c, remnant cholesterol and non-HDL-c remained significant after adjustment for maternal pre-pregnancy BMI and diet. This suggests that lipid levels may have a certain level of stability; independent of these factors. Therefore, the second pathway we hypothesized on was that genetic inheritance is an important contributor to the association of gestational lipid levels with lipid levels in childhood. In healthy women, LDL-c levels above the 99th percentile have been found to be caused by unfavorable genotypes or mutations causing familiar hypercholesterolemia [31]. In addition, several studies have found specific genes affecting lipid profiles [32–34]. A study of Kathiresan et al. found that almost 50% of childhood total cholesterol, triglycerides, LDL-c, and HDL-c levels can be explained by genetic inheritance [35]. Unfortunately, in this study we were not able to test genetic inheritance. However, although our results point towards genetic inheritance as an important contributor, the possible effects of lifestyle cannot be entirely ruled out. As a third pathway, the prenatal environment including nutritional exposures, may also have a strong impact on the epigenome through DNA methylation, which may result in phenotypic consequences in the offspring as shown in animal models and humans [36–39]. As a fourth pathway, we hypothesized that our findings may be the result of intrauterine programming of the fetus [13], which might be partially mediated by epigenetics. In women with a placental syndrome, insufficient foetal growth and development may occur, resulting in an increased risk of cardiovascular disease later in life [40, 41]. Therefore, we performed the same analyses in a subset of mothers without placental syndromes to see if intrauterine programming may explain our associations, however this did not change our results (Additional file 1, Table S2). We therefore hypothesize that our results may be explained by genetic inheritance and to a lesser extent by lifestyle or pregnancy-related factors.

This study found that girls had a more atherogenic lipid profile than boys, which is in agreement with a large population-based cohort providing reference levels for lipids in children [42]. Notably, in this study the girls had lower levels of HDL-c than boys at the age of 6 and 10 years. A study by Dathan-Stumpf et al. of 2571 children showed a continuous increase in serum HDL-c for both sexes up to the age of 8 years [43]. In addition, boys have a decrease in their HDL-c levels by 10–12 years of age, resulting in higher HDL-c levels in girls than boys from 12 years of age onwards [10]. Interestingly, the sex difference in lipid levels is already found in cord blood since girls have higher levels of total cholesterol and HDL-c in cord blood than boys [44], which may possibly be explained by hormonal influence [45]. Another reason may be a difference in body composition, as we found a higher BMI in girls than boys at the age of 10 years. Girls tend to have more fat mass than boys, which is associated with higher lipid levels [46, 47]. Also, ethnicity could be a possible reason for lipid profile variation between boys and girls, however we found no differences in the distribution of sexes among European and non-European children (data not shown).

Increased levels of total cholesterol, triglycerides, LDL-c, non-HDL-c, and low HDL-c levels are associated with cardiovascular disease and mortality later in life [48]. In order to reduce the risk of cardiovascular disease and its clinical consequences in later life, a low lifetime risk must be achieved by preventing an unfavourable lipid profile and the development of other risk factors from early life onwards [48, 49]. Dietary and lifestyle modifications could bring multiple benefits, including an improved lipid profile [50]. In a large randomized trial, lipid levels of low-risk pregnant women were safely modified through dietary changes from gestational week 17–20 until birth [51]. The PREDIMED trial including 7447 participants (55–80 years of age, without cardiovascular disease) has also shown that following a Mediterranean diet supplemented with consumption of healthy fats from extra-virgin olive oil or nuts, reduces the relative risk of CVD with 30%, compared to a low-fat diet [52]. As dyslipidaemia and obesity often co-exist, dietary interventions during pregnancy may also be beneficial in reducing excessive gestational weight gain [53].

Current guidelines of the American Heart Association and guidelines of the European Heart Association do not recommend to measure lipid levels in early pregnancy [54, 55]. However, based on this study, we suggest that in addition to routine pregnancy care glucose measurements it may be meaningful to measure lipid levels in order to initiate dietary changes if necessary. It may be even more interesting to measure lipid levels preconceptional since the preconceptional period gives opportunities to prevent later risks. This may be beneficial for timely intervention, especially since women who attend preconception care and pregnant women are willing to improve their lifestyle [56, 57]. Lipid levels measured in early pregnancy and subsequent beneficial lifestyle changes may be seen as a window-of-opportunity since lifestyle changes may not only affect pregnancy outcomes, but also future health of women and children.

### Strengths and limitations

We had a prospective data collection from early pregnancy onwards and a large sample of 2695 women and children with lipid measurements 6 years after pregnancy, and additional lipid measurements of 1673 children at the age of 10 years. Having two time points of lipid measurements during childhood is unique. In addition to the traditional lipid levels, we also assessed non-HDL-c and remnant cholesterol measures in children, since remnant cholesterol and non-HDL-c have already been proven important risk factors of cardiovascular disease in adults [58, 59]. Blood samples were collected with a minimum fasting time of 30 minutes and are therefore non-fasting. Changes in lipid and lipoprotein levels are considered minimal in response to normal food intake [60, 61]. In addition, lipid levels will also differ between fasting measurements in the same individual [60]. Non-fasting lipid measurements may also be more applicable in clinics since it may be more difficult to measure pregnant women in a fasting state. Regarding dietary assessment, data from the FFQs are self-reported and memory based, and the diet quality score ranging from 0 to 15 is rather rough. However, FFQs are still widely used as the primary dietary assessment tool in epidemiological studies. We tested unmeasured confounding for the regression models, and found that our models are considered rather robust. However, physical activity prior to or in early pregnancy may be considered as a potential confounder. Unfortunately, we did not have information on this variable and therefore did not include this potential confounder. Generalizability of this study may be comprised due to non-response at follow-up and since the women in this study had a relatively low BMI, which indicates selection towards a healthy population. Although we included many lifestyle factors, residual confounding may still be present. Future studies are warranted to investigate the genetic contribution on maternal lipid levels in pregnancy and lipid levels of their offspring years later.

## Conclusion

The gestational lipid profile in early pregnancy is associated with the lipid profile of children years after pregnancy, independent of maternal BMI and diet. Monitoring of these gestational lipid levels may give a window-of-opportunity to start early interventions to decrease offspring’s lipid levels and possibly diminish their cardiovascular risk later in life.

## Supplementary Information

**Additional file 1: Table S1.** E-value of the association of maternal lipid profile in early pregnancy with their corresponding offspring lipid level at the age of 6 and 10 years. **Table S2.** Lipid profile in boys and girls 6 (*n* = 2692) and 10 years after pregnancy (*n* = 1673). **Table S3.** Association of maternal lipid profile in early pregnancy with their corresponding offspring lipid level at the age of 6 and 10 years in women without a placental syndrome in their index pregnancy. **Table S4.** Association of cut-off values of the maternal lipid profile in early pregnancy with their corresponding offspring lipid level at the age of 6 and 10 years.

**Additional file 2.** STROBE statement.

## Data Availability

Raw data were generated at Generation R. Derived data supporting the findings of this study are available from the secretary of Generation R by sending an email to secretariaat.genr@erasmusmc.nl.

## Abbreviations

BMI: Body mass index
CI: Confidence interval
CVD: Cardiovascular Disease
DBP: Diastolic blood pressure
DNA: Deoxyribonucleic Acid
FFQ: Food frequency questionnaire
HDL-c: High-density lipoprotein-cholesterol
LDL-c: Low-density lipoprotein-cholesterol
PREDIMED trial: Prevención con Dieta Mediterránea trial
SBP: Systolic blood pressure
SD: Standard deviation
SDS: Standard deviation scores
SGA: Small-for-gestational age
sPTB: Spontaneous preterm birth
