# Supplementary material for: Maternal lipid levels in early pregnancy as a predictor of childhood lipid levels: a prospective cohort study
Source: BMC Pregnancy Childbirth. 2022 Jul 23;22:588. doi: 10.1186/s12884-022-04905-7 (PMC9308255; doi:10.1186/s12884-022-04905-7)
Supplement: Supplementary file 1 — Additional file 1: Table S1. E-value of the association of maternal lipid profile in early pregnancy with their corresponding offspring lipid level at the age of 6 and 10 years. Table S2. Lipid profile in boys and girls 6 (n = 2692) and 10 years after pregnancy (n = 1673). Table S3. Association of maternal lipid profile in early pregnancy with their corresponding offspring lipid level at the age of 6 and 10 years in women without a placental syndrome in their index pregnancy. Table S4. Association of cut-off values of the maternal lipid profile in early pregnancy with their corresponding offspring lipid level at the age of 6 and 10 years. [file 12884_2022_4905_MOESM1_ESM.docx]

**SUPPLEMENTAL MATERIAL**

**Additional file 1, Table S1.** E-value of the association of maternal lipid profile in early pregnancy with their corresponding offspring lipid level at the age of six and ten years.

| **Exposure**  **Lipids in pregnancy** | **Outcome**  **Lipid levels of children six years (n = 2692)** | | |  |
| --- | --- | --- | --- | --- |
|  | **Maternal BMI model**  Beta (95% CI) | **E-value** | | **E-value of upper limit** |
| Total cholesterol, SDS | 0.21 (0.16; 0.26) | 8.99 | | 7.16 |
| Triglycerides, SDS | 0.14 (0.09; 0.20) | 13.76 | | 9.47 |
| LDL-c, SDS | 0.19 (0.14; 0.25) | 9.99 | | 7.46 |
| HDL-c, SDS | 0.23 (0.18; 0.28) | 8.17 | | 6.60 |
| Remnant cholesterol, SDS | 0.14 (0.09; 0.20) | 13.76 | | 9.47 |
| Non-HDL-c, SDS | 0.19 (0.14; 0.24) | 9.99 | | 7.81 |
|  |  |  | |  |
| **Lipids in pregnancy** | **Outcome**  **Lipid levels of children ten years (n = 1673)** | |  |  |
|  | **Maternal BMI model**  Beta (95% CI) | **E-value** | | **E-value of upper limit** |
| Total cholesterol, SDS | 0.26 (0.21; 0.32) | 7.16 | | 5.70 |
| Triglycerides, SDS | 0.14 (0.09; 0.20) | 13.76 | | 9.47 |
| LDL-c, SDS | 0.26 (0.21; 0.31) | 7.16 | | 5.91 |
| HDL-c, SDS | 0.28 (0.23; 0.33) | 6.60 | | 5.51 |
| Remnant cholesterol, SDS | 0.15 (0.09; 0.20) | 12.82 | | 9.47 |
| Non-HDL-c, SDS | 0.24 (0.19; 0.29) | 7.81 | | 6.36 |

Abbreviations: BMI, body mass index; CI, confidence interval; SDS, SD-scores; LDL-c, low-density lipoprotein cholesterol; HDL-c, high-density lipoprotein cholesterol. Values are regression coefficients reflecting the difference in childhood lipid level with 95% confidence interval derived from multiple linear regression analyses. Maternal BMI model: adjusted for maternal age at intake, gestational age at blood sampling, ethnicity, parity, smoking, educational level, and pre-pregnancy BMI. E-value was calculated according to the manuscript of VanderWeele et al[1].

**Additional file 1, Table S2.** Lipid profile in boys and girls six (n = 2692) and ten years after pregnancy (n = 1673).

| **Lipids levels** | **Boys**  **Six years**  (n = 1361) | **Girls**  **Six years**  (n = 1331) | **Boys**  **Ten years**  (n = 822) | **Girls**  **Ten years**  (n = 851) |
| --- | --- | --- | --- | --- |
| BMI, kg/m^2^ | 15.9 (14.0; 19.0)^A^ | 15.9 (13.9; 19.9) ^B^ | 16.8 (14.4; 22.5)^D^ | 17.1 (14.4; 23.3) |
| Total cholesterol, mmol/L | 4.15 (0.64)^A,C^ | 4.26 (0.64)^B^ | 4.26 (0.64) ^D^ | 4.35 (0.64) |
| Triglycerides, mmol/L | 0.92 (0.44; 1.96)^A,C^ | 0.97 (0.48; 2.08) ^B^ | 0.98 (0.46; 2.39) ^D^ | 1.02 (0.49; 2.26) |
| LDL-c, mmol/L | 2.32 (0.58)^A,C^ | 2.45 (0.58) ^B^ | 2.25 (0.57) ^D^ | 2.39 (0.59) |
| HDL-c, mmol/L | 1.37 (0.32)^A,C^ | 1.32 (0.30) ^B^ | 1.50 (0.35) ^D^ | 1.43 (0.32) |
| Remnant cholesterol, mmol/L | 0.42 (0.20; 0.89)^A,C^ | 0.44 (0.22; 0.95) ^B^ | 0.45 (0.21; 1.09) ^D^ | 0.46 (0.22; 1.03) |
| Non-HDL-c, mmol/L | 2.78 (0.62)^C^ | 2.95 (0.61) ^B^ | 2.76 (0.62) ^D^ | 2.92 (0.64) |

Abbreviations: LDL-c, low-density lipoprotein-cholesterol; HDL-c, high-density lipoprotein-cholesterol. Data are presented as mean (SD) for continuous variables with a normal distribution or as median (90% range) for continuous variables with a skewed distribution.

^A^ Paired sample t-test or Wilcoxon Signed rank test of *P* < .05 of boys at the age of six years with corresponding measurements boys at the age of ten years.

^B^ Paired sample t-test or Wilcoxon Signed rank test of *P* < .05 of girls at the age of six years with corresponding measurements girls at the age of ten years.

^C^ Student’s t-test or Mann-Whitney U test of *P* < .05 of boys at the age of six years with the corresponding measurement in girls at the age of six years.

^D^ Student’s t-test or Mann-Whitney U test of *P* < .05 of boys at the age of ten years with the corresponding measurement in girls at the age of ten years.

**Additional file 1, Table S3.** Association of maternal lipid profile in early pregnancy with their corresponding offspring lipid level at the age of six and ten years in women without a placental syndrome in their index pregnancy.

| **Exposure**  **Lipids in pregnancy** | **Outcome**  **Lipid levels of children six years (n = 2143)** | | |
| --- | --- | --- | --- |
|  | **Basic model**  Beta (95% CI) | **Maternal BMI model**  Beta (95% CI) | **Maternal diet model**  Beta (95% CI) |
| Total cholesterol, SDS | 0.21 (0.15; 0.27) | 0.21 (0.15; 0.27) | 0.21 (0.15; 0.27) |
| Triglycerides, SDS | 0.15 (0.09; 0.21) | 0.15 (0.09; 0.21) | 0.15 (0.10; 0.21) |
| LDL-c, SDS | 0.19 (0.13; 0.25) | 0.19 (0.14; 0.25) | 0.19 (0.14; 0.25) |
| HDL-c, SDS | 0.25 (0.19; 0.30) | 0.25 (0.19; 0.30) | 0.25 (0.19; 0.31) |
| Remnant cholesterol, SDS | 0.15 (0.09; 0.21) | 0.15 (0.09; 0.21) | 0.16 (0.10; 0.22) |
| Non-HDL-c, SDS | 0.18 (0.12; 0.24) | 0.18 (0.12; 0.24) | 0.18 (0.12; 0.24) |
|  |  |  |  |
| **Lipids in pregnancy** | **Outcome**  **Lipid levels of children ten years (n = 1348)** | | |
|  | **Basic model**  Beta (95% CI) | **Maternal BMI model**  Beta (95% CI) | **Maternal diet model**  Beta (95% CI) |
| Total cholesterol, SDS | 0.26 (0.21; 0.32) | 0.26 (0.21; 0.32) | 0.26 (0.20; 0.32) |
| Triglycerides, SDS | 0.13 (0.07; 0.19) | 0.13 (0.07; 0.19) | 0.13 (0.07; 0.19) |
| LDL-c, SDS | 0.27 (0.21; 0.32) | 0.26 (0.21; 0.32) | 0.26 (0.21; 0.32) |
| HDL-c, SDS | 0.29 (0.23; 0.35) | 0.28 (0.22; 0.34) | 0.28 (0.23; 0.34) |
| Remnant cholesterol, SDS | 0.14 (0.08; 0.20) | 0.13 (0.07; 0.19) | 0.13 (0.07; 0.19) |
| Non-HDL-c, SDS | 0.24 (0.19; 0.30) | 0.24 (0.18; 0.30) | 0.24 (0.18; 0.30) |

Abbreviations: BMI, body mass index; CI, confidence interval; SDS, SD-scores; LDL-c, low-density lipoprotein cholesterol; HDL-c, high-density lipoprotein cholesterol. Values are regression coefficients reflecting the difference in childhood lipid level with 95% confidence interval derived from multiple linear regression analyses. Basic model: adjusted for maternal age at intake, gestational age at blood sampling, ethnicity, parity, smoking and educational level. Maternal BMI model: basic model additionally adjusted for pre-pregnancy BMI. Maternal diet model: BMI model additionally adjusted for maternal diet.

**Additional file 1, Table S4.** Association of cut-off values of the maternal lipid profile in early pregnancy with their corresponding offspring lipid level at the age of six and ten years.

| **Exposure**  **Lipids in pregnancy** | **Outcome**  **Lipid levels of children six years**  **(n = 2692)** | **Outcome**  **Lipid levels of children ten years**  **(n = 1673)** |
| --- | --- | --- |
|  | **Maternal BMI model**  Beta (95% CI) | **Maternal BMI model**  Beta (95% CI) |
| Total cholesterol, ≥ 90^th^ percentile (5.95 mmol/L) | 0.30 (0.22; 0.38) | 0.37 (0.27; 0.47) |
| Triglycerides, ≥ 90^th^ percentile (2.07 mmol/L) | 0.12 (0.06; 0.18) | 0.18 (0.11; 0.26) |
| LDL-c, ≥ 90^th^ percentile (3.35 mmol/L) | 0.26 (0.19; 0.34) | 0.31 (0.22; 0.40) |
| HDL-c, ≤ 10^th^ percentile (1.34 mmol/L) | 0.12 (0.08; 0.10) | 0.16 (0.11; 0.22) |
| Remnant cholesterol, ≥ 90^th^ percentile (0.94 mmol/L) | 0.12 (0.06; 0.18) | 0.19 (0.11; 0.26) |
| Non-HDL-c, ≥ 90^th^ percentile (4.11 mmol/L) | 0.25 (0.17; 0.33) | 0.32 (0.22; 0.42) |

Abbreviations: BMI, body mass index; CI, confidence interval; LDL-c, low-density lipoprotein cholesterol; HDL-c, high-density lipoprotein cholesterol. Values are regression coefficients reflecting the difference in childhood lipid level with 95% confidence interval derived from multiple linear regression analyses. Maternal BMI model: adjusted for maternal age at intake, gestational age at blood sampling, ethnicity, parity, smoking, educational level, and pre-pregnancy BMI.
